# A Novel Unit-Based Personalized Fingerprint Feature Selection Strategy for Dynamic Functional Connectivity Networks

**DOI:** 10.3389/fnins.2021.651574

**Published:** 2021-03-22

**Authors:** Feng Zhao, Zhiyuan Chen, Islem Rekik, Peiqiang Liu, Ning Mao, Seong-Whan Lee, Dinggang Shen

**Affiliations:** ^1^School of Computer Science and Technology, Shandong Technology and Business University, Yantai, China; ^2^BASIRA Lab, Faculty of Computer and Informatics Engineering, Istanbul Technical University, Istanbul, Turkey; ^3^School of Science and Engineering, Computing, University of Dundee, Dundee, United Kingdom; ^4^Department of Radiology, Yantai Yuhuangding Hospital, Yantai, China; ^5^School of Biomedical Engineering, ShanghaiTech University, Shanghai, China; ^6^Shanghai United Imaging Intelligence Co., Ltd., Shanghai, China; ^7^Department of Artificial Intelligence, Korea University, Seoul, South Korea

**Keywords:** dynamic functional connectivity networks, resting-state functional magnetic resonance imaging, feature selection strategy, functional connectivity network, autism spectrum disorder

## Abstract

The sliding-window-based dynamic functional connectivity networks (SW-D-FCN) derive from resting-state functional Magnetic Resonance Imaging has become an increasingly useful tool in the diagnosis of various neurodegenerative diseases. However, it is still challenging to learn how to extract and select the most discriminative features from SW-D-FCN. Conventionally, existing methods opt to select a single discriminative feature set or concatenate a few more from the SW-D-FCN. However, such reductionist strategies may fail to fully capture the personalized discriminative characteristics contained in each functional connectivity (FC) sequence of the SW-D-FCN. To address this issue, we propose a unit-based personalized fingerprint feature selection (UPFFS) strategy to better capture the most discriminative feature associated with a target disease for each unit. Specifically, we regard the FC sequence between any pair of brain regions of interest (ROIs) is regarded as a unit. For each unit, the most discriminative feature is identified by a specific feature evaluation method and all the most discriminative features are then concatenated together as a feature set for the subsequent classification task. In such a way, the personalized fingerprint feature derived from each FC sequence can be fully mined and utilized in classification decision. To illustrate the effectiveness of the proposed strategy, we conduct experiments to distinguish subjects diagnosed with autism spectrum disorder from normal controls. Experimental results show that the proposed strategy can select relevant discriminative features and achieve superior performance to benchmark methods.

## Introduction

The resting-state functional Magnetic Resonance Imaging (rs-fMRI), as a non-invasive neuroimaging technique, has been widely applied to capture the blood-oxygen-level-dependent (BOLD) signal which is sensitive to the spontaneous and intrinsic neural activity within the brain (Liu et al., 2008). In particular, the functional connectivity (FC), which is defined as the temporal correlation of the rs-fMRI signals between different brain regions of interest [ROIs, which is often defined through image registration (Luan et al., 2008; Jia et al., 2010; Wu et al., 2011, 2013)], can reflect the degree to which ROIs co-interact (Van Den Heuvel and Hulshoff Pol, 2010; Biao et al., 2016). A functional connectivity network (FCN) is usually represented as a graph, where each node represents a brain ROI and the edge between two nodes encodes the associated FC. Nowadays, many FCN models have been developed, from the simple conventional FCN (Zhang et al., 2015, 2016; Qiao et al., 2018) to the complex time-frequency analysis based dynamic FCN (Yaesoubi et al., 2015; Du et al., 2018), for diagnosing some neurodevelopmental diseases including the autism spectrum disorder (ASD) (Fornito et al., 2015; Liu et al., 2016; Huang et al., 2018; Zhao et al., 2018), the major depressive disorder (Greicius et al., 2007; Cullen et al., 2014) and so on.

Recent studies have shown that the dynamic changes of the correlation between ROIs contain abundant information (Damaraju et al., 2014; Wee et al., 2015). To explore the relationship between the dynamic changes and brain diseases while accounting for the time-varying connectivity patterns across ROIs, the sliding window based D-FCN (SW-D-FCN) is the most widely used technique on account of its simplicity and effectiveness (Chen et al., 2016, 2017; Du et al., 2018; Zhou et al., 2018). Specifically the sliding window approach is firstly used to generate a set of rs-fMRI subseries. Next, Pearson’s Correlation (PC) based temporal FCN is generated for each subseries. In the SW-D-FCN, the temporal change of the correlation between a pair of ROIs along the scanning time is defined as an FC sequence and thus SW-D-FCN also can be regarded as a set of FC sequences.

Feature extraction and selection are important in the process of exploring the time-varying connectivity patterns implicated in SW-D-FCN. Recently, many different feature extraction methods have been proposed. For example, Zhang et al. computed a weighted local clustering coefficient for each node in each network as a feature (Zhang et al., 2017); Chen et al. extracted features by calculating the root-mean-square for each FC sequence (Chen et al., 2017). Although many features can be obtained by various feature extraction methods to reflect the dynamic changes of an FC sequence, those features are prone to redundancy, which hinders the learning of robust classifiers. More importantly, many features may be irrelevant to the diagnosis of specific disease. Therefore, to improve the diagnosis performance, what kind of feature selection strategy should be adopted to select a smaller subset from the big feature set remains a key issue to solve.

Currently, there are two common feature selection strategies: the single-view based feature selection (SVFS) strategy and the concatenation-based feature selection (CFS) strategy.

**FIGURE 1:**
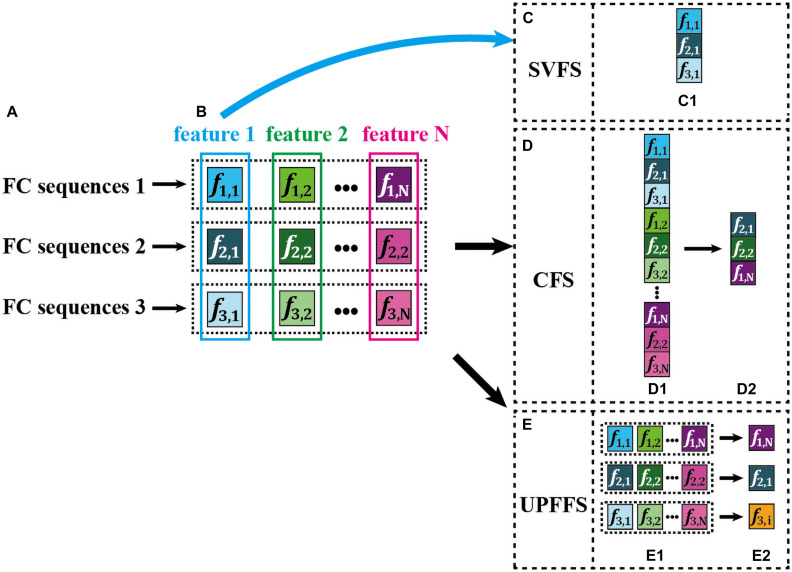
An intuitive explanation for three feature selection strategies. Here, the color saturation is used to encode the feature discriminative power. The higher the saturation, the more discriminative the features. Furthermore, the features with the highest color saturation are displayed in white text for easy viewing. **(A)** Three example FC sequences from D-FCN. **(B)** Multiple features extracted from each FC sequence, i.e., feature 1, feature 2… feature N. **(C)** Single-view based feature selection (SVFS) strategy. **(D)** Concatenation based feature selection (CFS) strategy. **(E)** The proposed unit-based personalized fingerprint feature selection (UPFFS) strategy.

SVFS selects one special set from multiple types of feature sets. For a better illustration, an example is displayed in Figure 1, where the feature 1 to feature N in Figure 1B represent N types of feature set, and the feature 1 is selected as the best feature set according to a given strategy (see Figure 1C). Due to its simplicity, this strategy has been widely applied in current studies. However, one type of feature set only reflects the characteristics from a single view, ignoring the personalized differences between FC sequences, thus could not comprehensively capture the subtle damage of brain functional tissues caused by neuropsychiatric diseases.

**FIGURE 2:**
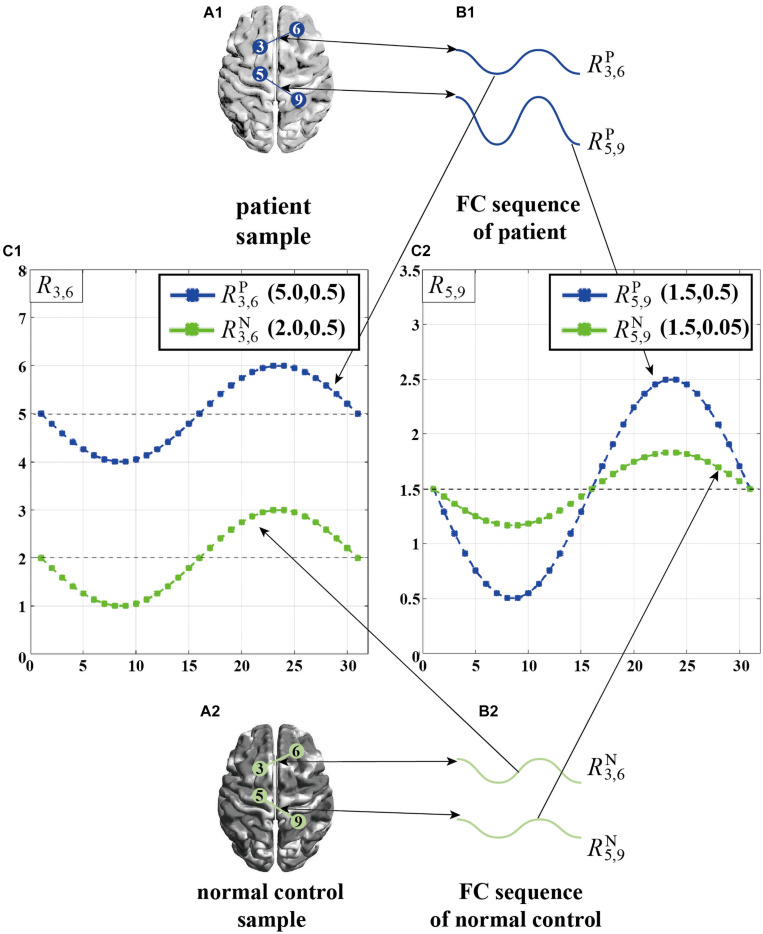
A simple example of the individual difference in the manifestation of connectivity differences between brain ROI pairs. **(A1)** Patient sample. **(A2)** Normal control sample. **(B1)** Two FC sequences from patient sample, ${\text{R}}_{3,6}^{P}<cps:it>and</cps:it>R_{5,9}^{P}$. **(B2)** Two FC sequences from normal control sample, ${\text{R}}_{3,6}^{N}\,\text{and}\,{\text{R}}_{5,9}^{N}$. **(C1)** Display of ${\text{R}}_{3,6}^{P}\,\text{and}\,{\text{R}}_{3,6}^{N}$. **(C2)** Display of ${\text{R}}_{5,9}^{P}\,\text{and}\,{\text{R}}_{5,9}^{N}$.

We further give a simple example in Figure 2, where $R_{3,6}^{P}$ denotes the FC sequence between the 3-rd ROI and the 6-th ROI of a given patient, $R_{3,6}^{N}$ denotes the FC sequence between the 3-rd ROI and the 6-th ROI of a normal control, and the values in the brackets denote the mean and variance of each FC sequence. As can be seen from Figure 2C1, in *R*_*3,6*_ (i.e., FC sequences between 3-rd ROI and 6-th ROI), the mean displays significant differences between the patient and the normal control, while the variance has no significant differences. On the other hand, in *R*_*5,9*_, as shown in Figure 2C2, the opposite is true, i.e., the variance largely varies between the patient and the normal control, while the mean has no significant differences. On one hand, solely using the mean as the inputting feature for subsequent classification will not fully tap into the discriminative ability of *R*_*5,9*_ since the mean of *R*_*5,9*_ is the same in both the patient and the normal control. On the other hand, we notice that only using the variance does not fully characterize the discriminative ability of *R*_*3,6*_. Therefore, SVFS methods might fail in fully utilized the discriminative power implied in part of FC sequences.

CFS, as an alternative feature selection and aggregation strategy, concatenates many types of feature set and inputs the resulting set to a target classifier for training. Figure 1 displays an illustration of this technique. Specifically, all features extracted by various feature extraction methods (Figure 1B) are concatenated into a long feature vector (Figure 1D1). Next, a feature evaluation method is used to select a few discriminative features (Figure 1D2), thereby forming a feature vector with a smaller size for the subsequent classification. However, the main drawback of CFS is that it may result in a sequence selection where for a few FC sequences, many types of features are selected for the subsequent classification, in contrast, for other FC sequences, not any features are selected. As can be seen in Figure 1D2, two types of features (i.e., *f*_*2,1*_ and *f*_*2,2*_) are selected from FC sequence 2, while no features of FC sequence 3 are included. Such strategy may lead to the issue that the selected smaller feature vector contains some redundant information due to the fact that many selected features as they are derived from the same FC sequence. Besides, such unbalanced selection by overlooking the diversity of features across all FC sequences may result in the inability to fully explore the discriminative information of each FC sequence.

It is worth noting that, brain diseases can cause abnormal connections among many pairs of ROIs due to the damage of brain. We believe that the manifestations of connectivity differences among different pair of ROIs are distinctive due to their distinct working mechanisms (Smith et al., 2011), which causes the discriminative fingerprint features from different FC sequences to exist huge difference. As explained above (Figure 2), the mean of *R*_*3,6*_ is more discriminative but its variance is worse, while the variance of *R*_*5,9*_ is more discriminative but its mean is worse, which indicates the mean can reflect the personalized connection characteristics of the 3-rd and 6-th ROIs, and the variance can reflect the personalized connection characteristics of the 5-th and 9-th ROIs. However, as previously described, both traditional CFS and SVFS feature selection strategies cannot fully explore the personalized feature of each FC sequence for more accurate classification of brain diseases.

To address the above issue, we propose a novel unit-based personalized fingerprint feature selection (UPFFS) strategy. Specifically, the FC sequence between any pair of ROIs is regarded as a unit which is handled independently. By using feature extraction methods, we extract multi-view features jointly from each unit, such as feature 1, feature 2, …, and feature N as shown in Figure 1B. Next, for each unit, the most discriminative one, which is regarded as its fingerprint feature, is selected from multi-view features by using a specific feature evaluation method, as shown in Figures 1E1,E2. Last, all the selected features are concatenated as a final feature set for the downstream classification task.

In summary, the main advantages of the proposed strategy are twofold: (1) For different FC sequences, the most discriminative feature types were are distinctive. The proposed UPFFS strategy can better extract personalized features according to the characteristic of each FC sequence, thus solving the single-view problem of SVFS; (2) due to the global damage of disease to human brain, the UPFFS can fully explore the damage information contained in each ROI pair, thereby avoiding the redundant information of CFS caused by the selection bias of the FC sequence, and while fully exploring the discriminative information of each FC sequence.

The rest of this article is organized as follows. In the “Materials and Methods” section, we introduce two baseline strategies (i.e., SVFS and CFS) and provide the details of our proposed unit-based personalized fingerprint feature selection (UPFFS) strategy. In the “Experiment and Results” section, we detail our experimental setting, including network construction, feature selection, classifier construction, and the evaluation measures. In the “Discussion” section, we discuss the impact of different feature evaluation methods, compare our strategy with other benchmarks, and analyze the identified discriminative features. Finally, the “Conclusion” section summarizes the paper.

## Materials and Methods

**FIGURE 3:**
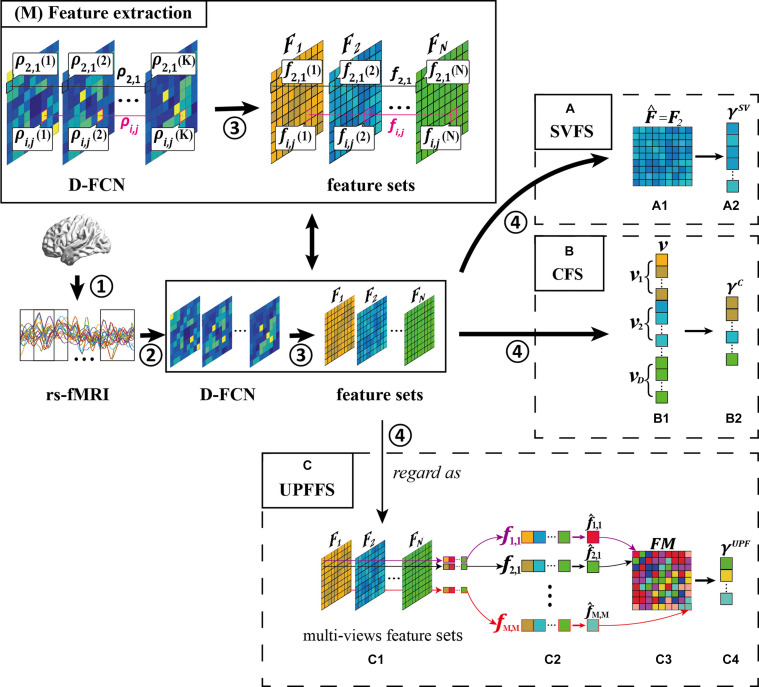
Overview of feature selection strategy, including four main steps: **①** rs-fMRI acquisition, **②** D-FCN construction, **③** feature extraction, and **④** feature selection. (M) Feature extraction. What’s more, we display three feature selection strategies: **(A)** single-view based feature selection (SVFS) strategy, **(B)** concatenation based feature selection (CFS) strategy; and **(C)** unit-based personalized fingerprint feature selection (UPFFS) strategy.

Figure 3 illustrates the flow of these three strategies (i.e., SVFS, CFS, and the proposed UPFFS) which will be described in detail in the following subsections. We also display the feature processing pipeline prior to feature selection while the detail of the feature processing will be introduced in the “Experiment and Results” section.

For ease of description, we define the variables that will be involved in the following subsections: (1) ρ_*i*,*j*_(*k*) denotes the correlation between the *i*-th ROI and the *j*-th ROI in *k*-th time window; (2) ρ_*i*,*j*_ = {ρ_*i*,*j*_[*cpsbreak*](1),ρ_*i*,*j*_(2),…,ρ_*i*,*j*_(*K*)}[*cpsbreak*](*i*,*j* = 1,2,…,*M*) denotes the FC sequence between the *i*-th ROI and the *j*-th ROI where *M* is the number of ROIs and *K* is the number of time windows; (3) *f*_*i*,*j*_(*d*) denotes the feature extracted from ρ_*i*,*j*_ with the *d*-th feature extraction method; (4) **F**_*d*_ = [*f*_*i*,*j*_(*d*)]_1 < *i*,*j* < *M*_(*d* = 1,2,3,…,*D*) aggregates all *d*-th features in ρ_*i*,*j*_ across all pairs of ROIs *i* and *j*.

### Traditional Feature Selection Strategies

#### Single-View Based Feature Selection Strategy

The main idea of single-view based feature selection (SVFS) is to select the best-performing type of feature set for the target classification task. Specifically, SVFS firstly tests the performance of each feature set **F**_*d*_ with a specific criterion (*d* = 1,2,…,*D*, where *D* is the number of feature extraction methods as motioned above), such as the classification accuracy, and then the best-performing feature set $\hat{\text{F}}$ is selected according to the following definition:

(1) $$\hat{F}=\text{opt}\,{\left\{F_{d}\right\}}_{d=1}^{D}$$

where $\text{opt}\,{\left\{{\text{F}}_{d}\right\}}_{d=1}^{D}$ denotes the selection of the best one from all types of feature sets.

Next, in order to remove the redundant features and preserve the discriminative features that are most likely relevant to disease, SVFS utilizes the feature evaluation method (e.g., the two-sample t-test between normal control and patient subjects) in the next step and the features with better evaluation results are retained to form the final feature set γ^**SV**^.

Figure 3A shows an example of SVFS. In this example, feature set 2 is selected as the optimal feature set in Figure 3A1, (i.e., $\hat{\text{F}}={\text{F}}_{2}$). Then, in Figure 3A2, the discriminative features are selected to form the final feature set γ^**SV**^.

In this way, the feature set that are most between-class discriminative can be selected. However, the personality differences of each FC sequence are ignored which may hinder the identification of the disease-altered brain connectivity patterns in a comprehensive manner, because some FC sequences may show discrimination information in other feature sets (e.g., **F**_1_,**F**_3_,…,**F**_*D*_) instead of **F**_2_.

#### Concatenation Based Feature Selection Strategy

The main idea of Concatenation Based Feature Selection (CFS) is to firstly concatenate many types of features as a big feature set and then filter out some discriminative features as the final feature set for classification. Specifically, in the first step, the features from each feature set **F**_*d*_ (*d* = 1,2,…,*D*) are vectorized as a vector **v_d_**. Next, all **v_d_** are concatenated into a long vector **v** and the discriminative features are selected from **v** with the feature evaluation method to form the final feature set γ^**C**^.

Figure 3B is an example of CFS where the vectors **v_d_** from each feature set **F_d_** are concatenated into a long vector in Figure 3B1, and in Figure 3B2, the discriminative features are selected to form the final feature set γ^**C**^.

Therefore, the discriminative information from all feature sets can be explored in such a way. However, the information redundancy that may be caused by the selected features in the same FC sequence and the omission of some FC sequences will affect the classification results.

### Unit-Based Personalized Fingerprint Feature Selection Strategy

In this subsection, we introduce a novel unit-based personalized fingerprint feature selection (UPFFS) strategy, which takes an adaptive approach in extracting the personalized fingerprint feature for each FC sequence.

Specifically, a FC sequence ρ_*i*,*j*_ = {ρ_*i*,*j*_(1),ρ_*i*,*j*_(2),…,ρ_*i*,*j*_(*K*)}(*i*,*j* = 1,2,…,*M*) is regarded as a unit and the multiple types of features from a unit are combined as a feature vector **f**_*i*,*j*_ (i.e., **f**_*i*,*j*_ = {*f*_*i*,*j*_(1),*f*_*i*,*j*_(2),…,*f*_*i*,*j*_(*K*)} where *f*_*i*,*j*_(1) denotes the first type of feature from ρ_*i*,*j*_), as shown in Figure 3C1. Next, the feature evaluation method is performed for each **f**_*i*,*j*_ internally. In this way, the most discriminative personalized fingerprint feature ${\hat{f}}_{i,j}$ (Figure 3C2) with the best evaluation result will be selected from each unit using the following formula:

(2) $${\hat{f}}_{i,j}=\text{opt}\,{\left\{f_{i,j}\,\left(d\right)\right\}}_{d=1}^{D}$$

where the$\mathrm{opt}\,{\left\{f_{i,j}\,\left(d\right)\right\}}_{d=1}^{D}$ denotes the selection of the best evaluation result from *f*_*i*,*j*_.

For the whole D-FCN, the fingerprint feature matrix **FM** (Figure 3C3) can be composed with the fingerprint features ${\hat{f}}_{i,j}$ of all FC sequences according to the following definition:

(3) $$\text{FM}={\left[{\hat{f}}_{i,j}\right]}_{1<i,j<M}$$

The features with better evaluation results than the default threshold will also be preserved to form a final fingerprint feature set γ^**UPF**^ (Figure 3C4) to avoid the impact of units which are less relevant to the disease.

This approach can not only solve the single-view problem of SVFS by considering multiple feature sets simultaneously, but also fully take into account the personalized features of each FC sequence which avoids the feature selection imbalance in CFS.

## Experiment and Results

To evaluate the effectiveness of the framework, we perform classification on the ASD subjects and normal controls. Specifically, we construct the traditional SW-D-FCN which is also called low-order dynamic functional connectivity networks (Lo-D-FCN), and the SW-D-FCN based high-order dynamic functional connectivity networks (Ho-D-FCN) on the rs-fMRI data set from the Autism Brain Imaging Data Exchange (ABIDE) database (Di Martino et al., 2014), apply seven feature extraction methods based on the central moment method, and then input the extracted features into a linear support vector machine (SVM) for linear fusion. In particular, the ASD data set and data preprocessing are same as those in a recent study (Wee et al., 2016).

### Comparison of Feature Selection Strategies Based on Lo-D-FCN

**FIGURE 4:**
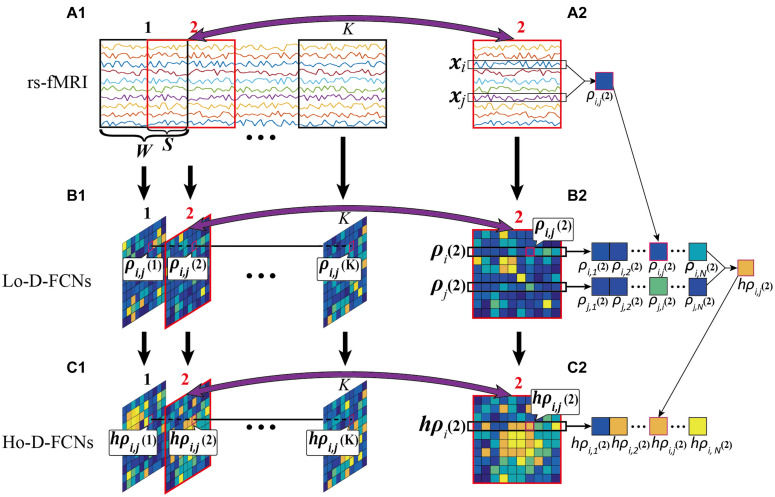
Flow chart of constructing Lo-D-FCN and Ho-D-FCN, where **(A1)** is the rs-fMRI series, **(A2)** is the second rs-fMRI subseries based on a sliding window, **(B1)** denotes the Lo-D-FCNs, **(B2)** denotes the second subnetwork of Lo-D-FCNs, **(C1)** denotes the Ho-D-FCNs, and **(C2)** denotes the second subnetwork from Ho-D-FCNs.

**Lo-D-FCN Construction.** In the construction of Lo-D-FCN, the entire rs-fMRI time series are firstly divided into *K* overlapping sub-windows by a sliding window with a prefixed window length *W*and a step size *S* (Figure 4A1). Next, for each sub-window, the correlations between the rs-fMRI time series from each pair of ROIs are calculated to create the sub Lo-FCN. As the example shown in Figures 4A2–B2, in the sub-window 2, **x_i_** and **x_j_** denote the rs-fMRI time series from the *i*-th and the *j*-th ROI respectively, and ρ_*i*,*j*_(2), as a component of sub Lo-FCN 2 (i.e., the second subnetwork of Lo-D-FCNs), denote the Pearson’s correlation (PC) of **x_i_** and **x_j_**. Repeating the above process, a series of sub Lo-FCN can be constructed which is called Lo-D-FCN (Figure 4B1). Obviously, the Lo-D-FCN can quantify the correlation change between each pair of ROIs in total scanning time.

**Feature Extraction.** In this experiment, we utilized seven center-distance feature extraction methods for extracting the dynamic variation of FC among multiple ROIs along the scanning time (Zhao et al., 2020). In particular, for Lo-D-FCN, the FC sequence of the *i*-th and the *j*-th ROI are defined as ρ_*i*,*j*_ = [ρ_*i*,*j*_(1),ρ_*i*,*j*_(2),…,ρ_*i*,*j*_(*K*)]. ρ_*i*,*j*_ reflects the FC dynamic changes of the *i*-th and the *j*-th ROI along the scanning timeline, which can be quantified by calculating its center-distance features. The features from all ρ_*i*,*j*_ can form a series of feature matrices.

**Classifier Learning.** The important features are selected using the traditional SVFS, CFS, and the proposed UPFFS strategies respectively from the set of all feature matrices. It is noteworthy that we utilize the t-test as the feature evaluation method in this experiment. Besides, we employ a sixfold cross-validation (CV) strategy to perform experiments and train the linear support vector machines (SVMs) (Cortes and Vapnik, 1995) for ASD classification tasks. For instance, one subject is selected as the testing dataset for each CV, while the other 5 subsets are used as the training dataset, and further, the nested fivefold CV is utilized on these 5 subsets to adjust the p-value in t-test model, as well as gamma (γ) and alpha (α) in SVM (classification learning and fusion parameters). In particular, the type of most discriminative feature of each FC sequence in UPFFS is determined with the training dataset.

For fair comparison, the sixfold CV was repeated 10 times and the samples are randomly partitioned each time differently to reduce the bias due to random seed selection and the optimal parameters are limited in the following range: p -value ∈ [0.01:0.01:0.1], γ ∈ [2^−5^,2^−4^,…,2^5^] and α ∈ [0.1:0.1:0.9].

**Classification Results.** Similar to previous studies (Zhao et al., 2017), we use classification accuracy (ACC), sensitivity or true positive rate (TPR), specificity or true negative rate (TNR), positive predictive value (PPV), negative predictive value (NPV), F1 score^1^ as performance indicators to quantify the performance of different feature selection strategies. Notably, in the classification work of this experiment, we consider ASD patients as positive and normal controls as negative.

Table 1 reports the best results of ASD identification with SVFS, CFS, and our UPFFS strategy in Lo-D-FCN where SVFS_*Lo*_ denotes the classification model using Lo-D-FCN and SVFS strategy. Specifically, for Lo-D-FCN construction, we select *W* = 60, *S* = 2 (e.g., the sliding-window length *W* equals to 60 and step size *S* equals to 2) as the optimal construction parameters according to the previous study (Zhao et al., 2020). The best results are highlighted in bold.

**TABLE 1:** ASD classification results with different feature selection strategies using Lo-D-FCN.

Model	ACC (%)	TPR (%)	TNR (%)	PPV (%)	NPV (%)	F1 (%)
SVFS_*Lo*_	70.4 ± 0.14	70.0 ± 0.45	70.8 ± 0.15	69.7 ± 0.11	71.4 ± 0.55	69.7 ± 0.20
CFS_*Lo*_	70.9 ± 0.15	69.6 ± 0.48	72.1 ± 0.23	70.5 ± 0.14	71.5 ± 0.21	69.9 ± 0.22
UPFFS_*Lo*_	**73.6 ± 0.06**	**70.7 ± 0.12**	**76.4 ± 0.11**	**74.2 ± 0.09**	**73.2 ± 0.06**	**72.3 ± 0.07**
						

From Table 1, we note that: (1) The proposed UPFFS strategy achieves better results than when using one of the seven center-distance methods alone (SVFS) or simple concatenation features (CFS); (2) the CFS just marginally boosts the classification accuracy compared with SVFS. The possible reason for such results is the unbalanced selection of the features from different FC sequences as previously mentioned in the “Introduction” section, and the UPFFS achieves better results by combining multiple types of features while avoiding this problem.

### Comparison of Feature Selection Strategies Based on Ho-D-FCN

**Ho-D-FCN Construction.** To further study the impact of different SW-D-FCN artifacts on the classification performance, we applied the above three strategies on the “correlation’s correlation” (Morris and Rekik, 2017; Soussia and Rekik, 2018) based Ho-D-FCN which is constructed by the sub Ho-FCN calculated from each sub Lo-FCN to extract higher-level interactions between ROIs. Specifically, in each Lo-FCN the correlation between one ROI and all other ROIs is regarded as a sequence, and the correlation between these sequences is calculated to form a Ho-FCN.

Figures 4B2–C2 shows an example of constructing the sub Ho-FCN 2 where ρ_*i*_(2) denotes the sequence of the correlations between the *i*-th ROI and the other ROIs in sub-window 2, and *h*ρ_*i*,*j*_(2), as a component of this sub Ho-FCN, denotes the correlation of ρ_*i*_(2) and ρ_*j*_(2). Repeating the above process, as shown in Figure 4C1, a series of sub Ho-FCN can be constructed which is called Ho-D-FCN. Obviously, the Ho-D-FCN can reflect the interaction pattern of temporal correlations from different ROI pairs over the whole scan time.

**Classification Results.** For Ho-D-FCN construction, we select *W* = 60, *S* = 10 as the construction parameters according to the previous research (Zhao et al., 2020). Since feature extraction and classifier learning are consistent with those above, we will not repeat them here. Table 2 displays the classification results where SVFS_*Ho*_ denotes the classification model with Ho-D-FCN and SVFS strategy. The best results are also highlighted in bold.

**TABLE 2:** ASD classification results with different feature selection strategies using Ho-D-FCN.

Model	ACC (%)	TPR (%)	TNR (%)	PPV (%)	NPV (%)	F1 (%)
SVFS_*Ho*_	70.7 ± 0.04	71.1 ± 0.12	70.2 ± 0.05	69.6 ± 0.03	71.8 ± 0.06	70.3 ± 0.05
CFS_*Ho*_	71.5 ± 0.17	71.3 ± 0.38	71.7 ± 0.14	70.7 ± 0.14	72.5 ± 0.24	70.9 ± 0.22
UPFFS_*Ho*_	**74.0± 0.10**	**73.8 ± 0.25**	**74.3± 0.12**	**73.3± 0.09**	**74.9± 0.15**	**73.5± 0.13**
						

It can be seen that the results of Ho-D-FCN are similar to those of Lo-D-FCN despite the modest improvement in accuracy. Specifically, the proposed UPFFS performs significantly better than these two traditional strategies in Ho-D-FCN, and CFS also slightly improves the classification performance compared with SVFS, which further supports our preliminary hypothesis.

### Classification Performance Based on the Fusion of Lo-D-FCN and Ho-D-FCN

There are complex connections inside the brain, thus it is difficult to fully capture the relationship between various regions through a single type of FCN. In order to further improve the classification performance, it is a general practice to combine the Lo-D-FCNs and Ho-D-FCNs by linear fusion of SVM ensemble decision scores (Zhao et al., 2020). In this paper, we fuse the SVM scores from two D-FCNs using different feature selection strategies. Specifically, each SVM classifier can output a decision score indicating the probability of a subject belonging to a class. So we calculate the weighted average of the decision scores from two SVM classifiers to obtain the classification results, where the weight tuned for each SVM is determined using a nested CV. The final classification results are shown in Table 3, where the symbol “ + ” represents the fusion, for example, SVFS_*Lo*_ + SVFS_*Ho*_ represents the fusion of Lo-D-FCN and Ho-D-FCN under the SVFS. We also highlight the best results with a bold font in Table 3.

**TABLE 3:** Fusion result of different feature selection strategies.

Model	ACC (%)	Promote (Lo/Ho)	TPR (%)	TNR (%)	PPV (%)	NPV (%)	F1 (%)
SVFS_*Lo*_ + SVFS_*Ho*_	71.4 ± 0.23	1.0/0.7	73.1 ± 0.16	69.8 ± 0.14	69.9 ± 0.10	73.1 ± 0.12	71.4 ± 0.11
CFS_*Lo*_ + CFS_*Ho*_	72.8 ± 0.07	1.9/1.3	72.0 ± 0.36	73.6 ± 0.14	72.3 ± 0.06	73.5 ± 0.14	72.1 ± 0.13
UPFFS_*Lo*_ + UPFFS_*Ho*_	**76.0 ± 0.10**	**2.4/2.0**	**74.4 ± 0.31**	**77.5 ± 0.20**	**76.0 ± 0.13**	**76.1 ± 0.14**	**75.1 ± 0.14**

In addition to the indicators above, we also display the improvement of fusion results compared to the single D-FCN in classification accuracy with the Promote(Lo/Ho) indicator (e.g., 2.4/2.0 represents that the fusion network improved 2.4 compared to Lo-D-FCN and 2.0 compared to Ho-D-FCN in the classification accuracy). Based on Table 3, we derive the following conclusions: (1) the feature fusion result consistently produces better results than features derived from a single network, i.e., the promote indicators are all greater than 0, which is similar to the conclusions of previous studies (Zhao et al., 2020); (2) our UPFFS strategy has better results and greater improvement compared with other strategies, which indicates that our feature selection framework can select more complementary discriminative features from Lo-D-FCN and Ho-D-FCN, thus the SVM classifier has a better learning and generalizability to unseen samples.

## Discussion

### Effect of Different Feature Evaluation Method

**FIGURE 5:**
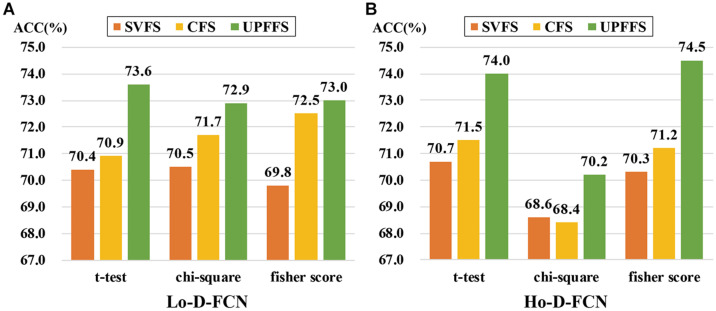
Classification performance comparison among different feature evaluation methods. **(A)** The result of Lo-D-FCN. **(B)** The result of Ho-D-FCN.

The feature evaluation method is an important part of our UPFFS strategy. In order to investigate the effect of the feature evaluation method on the classification performance, we further utilize chi-square and fisher score as our feature evaluation method respectively to replace the *t*-test, then perform classification experiments following the same steps. In particular, the threshold for alternative feature evaluation method is also adjusted by nested CV, where the threshold range of chi-square is set to [0.1:0.1:1] and the threshold range of fisher score is [0.01:0.005:0.05] according to previous studies. The experimental results are shown in Figure 5.

It can be seen from Figure 5 that UPFFS always has a better result than traditional strategies with different feature evaluation methods, which further shows the outperformance of the UPFFS strategy. On the other hand, we empirically found that the performances are sensitive to the feature evaluation methods. For instance, the ASD classification results of chi-square in Ho-D-FCN (Figure 5B) are lower than the others. Thus, in practice, the feature evaluation methods of UPFFS should be utilized carefully depending on the dataset in hand and the target classification task.

### Top Discriminative Feature

For exploring the internal reasons that the UPFFS strategy improves the ASD classification performance from a physiological aspect, we identify the set of the most discriminative features. Specifically, we count the frequency at which features are selected within the sixfold cross-validation and quantify the discriminative ability of one feature with its frequency. The higher its frequency, the more discriminative it is regarded.

**FIGURE 6:**
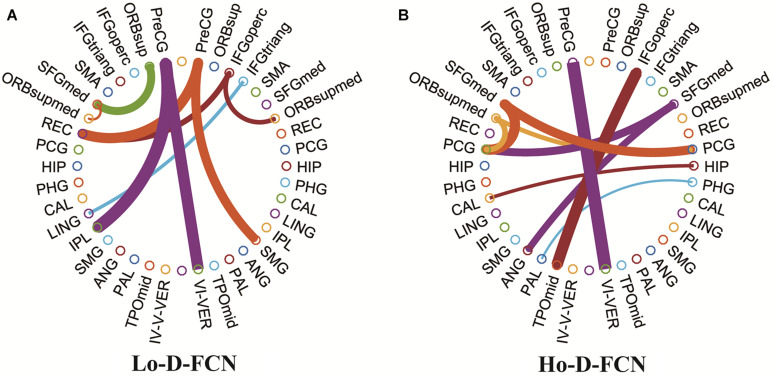
Circular graphs of the top 10 discriminative connections selected by UPFFS. **(A)** The Lo-D-FCNs. **(B)** The Ho-D-FCNs.

Figure 6 visualizes the top 10 most discriminative features selected by UPFFS strategy in Lo-D-FCN (Figure 6A) and Ho-D-FCN (Figure 6B) respectively with circular graphs, where a node denotes an ROI and a line between the two nodes denotes a connection feature which represents the correlation between two ROIs (Krzywinski et al., 2009). Table 4 lists the abbreviations of brain regions in Figure 6.

**TABLE 4:** Abbreviations of ROIs selected from Lo-D-FCN and Ho-D-FCN.

Abbreviation	ROI name	Abbreviation	ROI name
PreCG	Precentral gyrus	ROBsup	Orbitofrontal cortex (middle)
IFGoperc	Inferior frontal gyrus (opercular)	IFGtrian	Inferior frontal gyrus (triangular)
SMA	Suplementary motor area	SFGmed	Superior frontal gyrus (medial)
ORBsupmed	Orbitofrontal cortex (medial)	REC	Rectus gyrus
PCG	Posterior cingulate gyrus	HIP	Hippocampus
PHG	ParaHippocampal gyrus	CAL	Calcarine cortex
LING	Lingual gyru	IPL	Inferior parietal lobul
SMG	Supramarginal gyrus	ANG	Angular gyrus
PAL	Pallidum	TPOmid	Temporal pole (middle)
IV-V-VER	Lobule IV, V of vermis	VI-VER	Lobule VI of vermis

From the results shown in Figure 6 and Table 4, we find that: (1) the corresponding ROIs of the top discriminative connection features selected by the UPFFS strategy include the superior frontal gyrus, orbitofrontal cortex, hippocampus, calcarine cortex, angular gyrus, etc., which are associated with visual processing, social cognition, and emotional expression. These findings are consistent with previous studies (Di Martino et al., 2009; Scherf et al., 2015). In particular, superior frontal gyrus (medial) (Goldberg et al., 2006; Sato et al., 2012), orbitofrontal cortex (medial) (Ye et al., 2014), hippocampus (Ha et al., 2015), calcarine cortex (Libero et al., 2014; Perkins et al., 2015), angular gyrus (Goldberg et al., 2006) have been reported as potential biomarkers for ASD identification. (2) Most of the selected discriminative features connect the brain lobe of transhemisphere rather than the same hemisphere, which indicates that the UPFFS strategy can identify abnormal distribution patterns over the whole brain. (3) The discriminative features of Lo-D-FCNs, and Ho-D-FCNs are obviously different, indicating that these two networks can contain complementary information for ASD and that our UPFFS strategy is able to capture such rich and personalized information.

## Conclusion

In this paper, we proposed a new feature selection strategy, called UPFFS, which regarded the FC sequence between any pair of brain ROIs as a unit and extracts the personalized fingerprint feature from each unit, thus reflecting the discriminative information in SW-D-FCN more comprehensively. We evaluated the performance of the proposed UPFFS and two traditional approaches on two types of SW-D-FCN. The experimental results have shown that: (1) UPFFS always has better ability to extract discriminative information compared with the two traditional approaches; (2) UPFFS is able to extract features that are unique to the network, making features from different networks more complementary and causing a higher improvement in classification result for the fusion of two networks; (3) we also found that the top selected discriminative brain regions by UPFFS are related to visual processing, social cognition, and emotional expression which is in line with previous research results and further validates the effectiveness of UPFFS.

Although we proposed this strategy for SW-D-FCN, this idea can be utilized in other fields requiring personalized feature identification, and it should be indicated that the feature evaluation method have a certain influence on the classification results. In our future work, we will apply the UPFFS to other types of “omic” datasets such as genomics for underpinning biological biomarker in a variety of disorders.

## Data Availability Statement

The original contributions presented in the study are included in the article/supplementary material, further inquiries can be directed to the corresponding author/s.

## Author Contributions

All authors listed have made a substantial, direct and intellectual contribution to the work, and approved it for publication.

## Conflict of Interest

DS was a consultant of Shanghai United Imaging Intelligence Co., Ltd. The commercial affiliation did not play a role in the study design, data collection and analysis, decision to publish, or preparation of the manuscript. The remaining authors declare that the research was conducted in the absence of any commercial or financial relationships that could be construed as a potential conflict of interest.

## Footnotes

1 https://en.wikipedia.org/wiki/Sensitivity_and_specificity
